# Differences in the symptom experience of older versus younger oncology outpatients: a cross-sectional study

**DOI:** 10.1186/1471-2407-13-6

**Published:** 2013-01-03

**Authors:** Janine K Cataldo, Steven Paul, Bruce Cooper, Helen Skerman, Kimberly Alexander, Bradley Aouizerat, Virginia Blackman, John Merriman, Laura Dunn, Christine Ritchie, Patsy Yates, Christine Miaskowski

**Affiliations:** 1 School of Nursing, University of California, 2 Koret Way – N631Y, San Francisco, CA, 94143-0610, USA; 2 Schools of Nursing Medicine, University of California, 2 Koret Way – N631Y, San Francisco, CA, 94143-0610, USA; 3 School of Nursing, Queensland University of Technology, Victoria Park Road, Kelvin Grove, 4059, Queensland, Australia; 4 Institute for Human Genetics, University of California, 2 Koret Way – N631Y, San Francisco, CA, 94143-0610, USA; 5 Department of Physiological Nursing, University of California, 2 Koret Way – N631Y, San Francisco, CA, 94143-0610, USA

**Keywords:** Geriatric oncology, Symptoms

## Abstract

**Background:**

Mortality rates for cancer are decreasing in patients under 60 and increasing in those over 60 years of age. The reasons for these differences in mortality rates remain poorly understood. One explanation may be that older patients received substandard treatment because of concerns about adverse effects. Given the paucity of research on the multiple dimensions of the symptom experience in older oncology patients, the purpose of this study was to evaluate for differences in ratings of symptom occurrence, severity, frequency, and distress between younger (< 60 years) and older ( ≥ 60 years) adults undergoing cancer treatment. We hypothesized that older patients would have significantly lower ratings on four symptom dimensions.

**Methods:**

Data from two studies in the United States and one study in Australia were combined to conduct this analysis. All three studies used the MSAS to evaluate the occurrence, severity, frequency, and distress of 32 symptoms.

**Results:**

Data from 593 oncology outpatients receiving active treatment for their cancer (i.e., 44.4% were < 60 years and 55.6% were ≥ 60 years of age) were evaluated. Of the 32 MSAS symptoms, after controlling for significant covariates, older patients reported significantly lower occurrence rates for 15 (46.9%) symptoms, lower severity ratings for 6 (18.9%) symptoms, lower frequency ratings for 4 (12.5%) symptoms, and lower distress ratings for 14 (43.8%) symptoms.

**Conclusions:**

This study is the first to evaluate for differences in multiple dimensions of symptom experience in older oncology patients. For almost 50% of the MSAS symptoms, older patients reported significantly lower occurrence rates. While fewer age-related differences were found in ratings of symptom severity, frequency, and distress, a similar pattern was found across all three dimensions. Future research needs to focus on a detailed evaluation of patient and clinical characteristics (i.e., type and dose of treatment) that explain the differences in symptom experience identified in this study.

## Background

Cancer is predominantly an older person’s illness. Approximately 50% of the most common cancers (i.e., breast, prostate, and lung) occur in people over 60 years of age [1]. Because of major advances in cancer treatment and supportive care strategies, a larger percentage of older persons are receiving active treatment [2]. However, while mortality rates for cancer are decreasing in patients under 60, they are increasing in those over 60 years of age [1]. The reasons for these differences in mortality rates remain poorly understood primarily due to the paucity of research on older persons with cancer.

Older adults are a vulnerable population who are at high risk for suboptimal cancer care because they were under-represented in clinical trials and experience disparities in cancer treatment [3-7]. One reason why mortality rates may be increasing in patients over 60 is that they receive substandard treatment (i.e., lower doses of radiation therapy (RT) or chemotherapy (CTX)) because of the unsubstantiated belief that they will experience a larger number and more severe adverse effects as well as poor functional outcomes and significant decrements in quality of life (QOL) [8,9]. The foundations for these beliefs include the normal physiological changes that occur with aging that could influence drug metabolism, the co-occurrence of multiple co-morbidities in the elderly, and the increased risk of adverse effects associated with polypharmacy [2,10,11]. However, findings from studies on the impact of co-morbidities and cancer treatment on older people are contradictory [12-14].

In addition, in the limited number of studies that evaluated associations between age and the occurrence and severity of physical and psychological symptoms, the results are inconsistent. In a study of oncology patients undergoing RT [15], sleep disturbance, pain, and distress were significantly less prevalent among older patients compared to younger patients, while shortness of breath was significantly more prevalent among older patients before RT. In another study of newly diagnosed cancer patients [16], age was weakly correlated with ratings of symptom distress (*r* = −0.11, p < 0.02) and older patients reported lower levels of symptom distress. In contrast, in a study of multiple symptoms [17], patients over 70 reported higher symptom distress scores than patients less than 40 years of age. In a study of patients with advanced cancer [10], compared to older patients, younger patients reported higher pain severity scores and better appetite. In terms of psychological symptoms, older age is consistently associated with lower levels of psychological distress regardless of clinical factors [18-21]. However, less is known about how older patients experience psychological distress associated with cancer and its treatment [22,23].

In both Australia and the United States, the number of oncology patients over the age of 60 is growing exponentially [1,24]. In fact, estimates suggest that by 2030, the incidence of cancer among older adults will increase by 67% [25]. If research studies are not done to characterize the symptom management needs of older persons with cancer, the medical systems in both countries will not be prepared to provide the supportive care interventions that these highly vulnerable patients need in order to receive appropriate treatments and survive their cancer. Therefore, given the paucity of research on age-related differences in the multiple dimensions of the symptom experience, the purpose of this study, using the Memorial Symptom Assessment Scale (MSAS) [26], was to evaluate for differences in ratings of symptom occurrence, severity, frequency, and distress between younger (< 60 years) and older (≥ 60 years) adults undergoing cancer treatment. Based on previous studies [16,27-29], we hypothesized that older patients would have significantly lower ratings on all symptom dimensions than younger patients.

## Methods

### Study samples

Demographic, clinical, and symptom data from one Australian study (i.e., Symptom Clusters) and two United States studies (i.e., Fatigue, Pain, and Sleep Study (FPS study), Symptom Prevalence Study) were combined to conduct this analysis. All three studies enrolled patients who were receiving active treatment for their cancer. To evaluate the effect of age, patients were dichotomized into younger (< 60) and older (≥ 60) groups based on cut-offs used in previous studies of cancer symptoms [10,30,31], as well as findings that cancer mortality rates are increasing in those > 60 years [1].

#### Symptom clusters study

This prospective, longitudinal study was designed to identify symptom clusters and their effects on physical and psychological functioning of patients with metastatic disease. Data were collected from patients using an interview-administered survey at the time of diagnosis or progression of metastatic disease and again at 2 months and 4 months. Data from the first assessment were used in these analyses.

Patients were recruited consecutively from two major tertiary referral hospitals in Australia: the Royal Brisbane and Women’s Hospital and Peter MacCallum Cancer Centre (Melbourne). Patients were to participate if they: were adults (> 18 years of age) who could read, write, and understand English; had no cognitive limitations; had a primary cancer of breast, lung, colon/rectum, prostate, upper gastrointestinal tract, or ovaries; and were diagnosed with metastatic disease in the past month or had clinical evidence of progressive metastatic disease. Patients were excluded if they had local recurrence, but no evidence of metastatic disease; had a prognosis of < 4 months as determined by their clinician; or had physical or cognitive impairments that precluded participation in the 15-minute survey. The study was approved by the Ethics Committees of Queensland University of Technology and the two participating hospitals.

Research staff liaised with clinical staff to identify potentially eligible patients, using a standard screening assessment sheet. All patients provided written consent prior to completing the study questionnaires. Of 306 patients approached, 218 patients were recruited (71.2% response rate). Reasons for non-participation included: a clinician assessed that patients were not well enough to participate (27.9%), prognosis < 4 months (22%), limited English (18.7%), patient was currently participating in another study (18.4%), and physically or cognitively unable to participate (13%). The questionnaire was completed during a 20 minute face-to-face interview conducted by trained interviewers with nursing or psychology backgrounds. Clinical and demographic data were obtained from medical record reviews.

#### FPS study

This longitudinal study evaluated multiple symptoms in patients who underwent primary or adjuvant RT. Patients were recruited from two RT departments located in a Comprehensive Cancer Center and a community-based oncology program at the time of the patient’s simulation visit. Patients were eligible to participate if they: were ≥ 18 years of age; were scheduled to receive primary or adjuvant RT for one of four cancer diagnoses (i.e., breast, prostate, lung, brain); were able to read, write, and understand English; gave written informed consent; and had a Karnofsky Performance Status (KPS) score of ≥ 60. Patients were excluded if they had: metastatic disease, more than one cancer diagnosis, or a diagnosed sleep disorder.

A total of 472 patients were approached and 185 consented to participate (response rate of 34%). The primary reasons for refusal were being overwhelmed or too busy. The study was approved by the Committee on Human Research at the University of California, San Francisco (UCSF) and at the second site. At the time of the simulation visit (i.e., approximately one week prior to the initiation of RT), patients were approached by a research nurse to discuss participation in the study. After obtaining written informed consent, patients completed the study questionnaires. In addition, medical records were reviewed for disease and treatment information.

#### Symptom prevalence study

This descriptive, cross-sectional study used self-report questionnaires to obtain information from a convenience sample of oncology outpatients. Patients were recruited from four outpatient settings in Northern California, including a university-based Cancer Center, a Veterans Affairs facility, and two community-based outpatient clinics. Patients were eligible to participate if they were > 18 years of age; were able to read, write, and understand English; gave written, informed consent; had KPS scores of ≥ 50; and were receiving active cancer treatment.

A total of 310 patients were approached and 206 consented to participate (response rate of 66%). The primary reasons for refusal were that a patient was too ill to participate (80%), too busy (15%), or not interested in the research study (5%). Patients who agreed to participate provided written informed consent and were given a copy of the questionnaire booklet. They completed the study questionnaires in their home and returned them to the research office using a postage paid envelope. The study was approved by the Committee on Human Research at UCSF and at each of the study sites.

### Instruments

#### Demographic and clinical characteristics

Demographic information on age, gender, marital status, and living arrangements were obtained at enrollment. Because of differences in the educational systems in Australia and the United States, data on education were recoded into a dichotomous variable (i.e., no post high school versus post high school education). In addition, patients’ medical records were reviewed for cancer diagnosis, presence of metastatic disease, and current treatment regimens (i.e., none, CTX, RT, or both CTX and RT).

In the Australian study, patient’s functional status was rated by their clinician using the Eastern Cooperative Oncology Group (ECOG) Performance Status score that ranges from 0 (fully active) to 4 (disabled) [32]. In the United States studies, patients rated their functional status using the KPS scale that ranged from 30 (I feel severely disabled and need to be hospitalized) to 100 (I feel normal; I have no complaints or symptoms). The KPS scale is widely used to evaluate functional status in patients with cancer and has well established validity and reliability. Based on the recommendations of Verger and colleagues [33], the KPS scores were converted to ECOG scores for use in subsequent analyses.

#### Memorial Symptom Assessment Scale (MSAS)

All three studies used the MSAS to evaluate the occurrence, severity, frequency, and distress of 32 symptoms commonly associated with cancer and its treatment [26]. The MSAS is a self-report questionnaire designed to measure the multidimensional experience of symptoms. Using the MSAS, patients were asked to indicate whether or not they had experienced each symptom in the past week (i.e., symptom occurrence). If they had experienced the symptom, they were asked to rate its frequency of occurrence, severity, and distress. Symptom frequency was evaluated using a 4-point Likert scale (i.e., 1 = rarely, 2 = occasionally, 3 = frequently, 4 = almost constantly). Symptom severity was measured using a 4-point Likert scale (i.e., 1 = slight, 2 = moderate, 3 = severe, 4 = very severe). Symptom distress was measured using a 5-point Likert scale (i.e., 0 = not at all, 1 = a little bit, 2 = somewhat, 3 = quite a bit, 4 = very much). Three subscale scores (i.e., physical, psychological, global distress index) and a total MSAS score were calculated. The reliability and validity of the MSAS is well established in studies of oncology inpatients and outpatients [26,34]. Cronbach’s alphas for the total sample, as well as for patients < 60 years and ≥ 60 years are as follows: physical subscale (.82, .82, .82), psychological subscale (.81, .80, .77), global distress index (.82, .80, .83), and total MSAS score (.88, .89, .87).

### Analysis

The three data sets were combined and data were analyzed using SPSS version 19 and STATA SE Version 12. Descriptive statistics, means, and standard deviations for quantitative variables and frequencies and percentages for categorical variables were generated to describe various patient characteristics. Mean ratings of severity, frequency, and distress were calculated for those patients who reported the symptom. Because previous studies have dichotomized older and younger patients at age 60 [10,30,31], older patients were defined as adults ≥ 60 years of age. Independent sample t-tests and Chi Square analyses were used to evaluate for differences in demographic and clinical characteristics between the two age groups. Differences between the two age groups in characteristics with multiple levels (i.e., diagnosis, treatment, ECOG Performance Status score) were further examined using post-hoc contrasts with Bonferroni correction.

Logistic regression was used to evaluate the effect of increasing age on the occurrence rates of each symptom. Significant differences, between the two age groups (< 60 (younger) and ≥ 60 (older) years of age), in the occurrence rates of each symptom were evaluated with binary logistic regression analyses. To examine the differences between the two age groups in the severity, frequency, and distress ratings of each symptom, ordinal logistic regression was utilized. Significant differences, between the two age groups (< 60 (younger) and ≥ 60 (older) years of age), in the MSAS subscale and total scores were evaluated with linear regression analyses. For all of the regression analyses, unadjusted and adjusted values (e.g., odds ratios and their corresponding 95% confidence intervals) were generated for each symptom or scale score.

## Results

### Differences in demographic and clinical characteristics between older and younger patients

As shown in Table 1, data from 593 oncology outpatients (i.e., 44.4% were < 60 years and 55.6% were ≥ 60 years of age) were evaluated. No differences were found across the three studies, in the age distribution of the patients. In addition, no differences were found, across the three studies, in the mean age of the patients (i.e., Symptom Clusters Study = 62.2 (± 12.0) years, FPS Study = 60.8 (± 12.0) years, Symptom Prevalence Study = 60.8 (± 12.4) years; F(2,590) = .994, p = .371). Across the three studies, the age distribution in 10 year increments was: 20 to 29 = 0.8%, 30 to 39 = 4.2%, 40 to 49 = 12.0%, 50 to 59 = 27.3%, 60 to 69 = 29.0%, 70 to 79 = 21.4%, and ≥ 80 = 5.3%.

**Table 1:** **Demographic and clinical characteristics for the total sample and differences between younger** (<** 60**, **n** = **263**) **and older** (≥ **60**, **n** = **330**) **patients**

**Characteristic**	**Total(%)**	**Age group**		**p**-**value**	**Post hoc contrasts**		
		**< 60(%)**	**≥ 60(%)**		**< 60(%)**	**≥ 60(%)**	**p**-**value**
Study project							
Fatigue, Pain, and Sleep	28.8	28.9	28.8	.170			
Symptom Prevalence	34.4	38.0	31.5				
Symptom Clusters	36.8	33.1	39.7				
Gender- Female	54.6	70.7	41.8	< .001			
Lives alone	26.4	24.0	28.2	.260			
Partnered/married	60.9	57.3	63.7	.126			
Education – Post high school	61.4	68.8	55.5	.001			
Diagnosis							
Breast	33.6	48.3	21.8		48.3	21.8	.0125
Prostate	26.0	11.0	37.9	< .001	11.0	37.9	.0125
Lung	13.2	11.4	14.5				
Other	27.3	29.3	25.8				
Metastases	34.9	37.3	32.9	.297			
Treatment							
None	15.5	13.3	17.3				
Only radiation	43.6	33.8	51.4	< .001	33.8	51.4	.0125
Only chemotherapy	27.4	31.2	24.3				
Both	13.5	21.7	7.0		21.7	7.0	.0125
ECOG Performance Status							
Fully active	21.4	15.8	26.0		15.8	26.0	.0100
Ambulatory, light work	48.9	53.1	45.5	.043			
Ambulatory, mobile > 50%	21.6	23.5	20.1				
Ambulatory, mobile < 50%	7.2	6.5	7.7				
Disabled	0.9	1.2	0.6				
Age (years; mean (standard deviation))	61.3 (12.1)	50.3 (7.7)	70.1 (6.5)	< .001			

Abbreviation: ECOG, Eastern Cooperative Oncology Group.

The mean age of the entire sample was 61.3 (±12.1) years. On average, younger patients were 50.3 (±7.7) and older patients were 70.1 (±6.5) years of age (p < .001). Older patients were: more likely to be male (p < .001); less likely to have finished high school; more likely to have prostate cancer (p < .001); more likely to be receiving RT (p < .001), and more likely to be fully active (p = .04).

### Differences in symptom occurrence rates between older and younger patients

Table 2 provides the symptom occurrence rates for the total sample for each of the MSAS symptoms, as well as the unadjusted and adjusted odds of symptom occurrence as age increases by one year. After controlling for significant covariates (i.e., gender, education, diagnosis, treatment, and ECOG performance status), as age increased, significantly lower occurrence rates were reported for difficulty concentrating, pain, feeling nervous, nausea, difficulty sleeping, feeling bloated, vomiting, feeling sad, sweats, worrying, problems with sexual interest, feeling irritable, I don’t look like myself, and changes in skin.

**Table 2:** Symptom occurrence rates for the total sample and odds of symptom occurrence as age increases by one year

**Symptom**	**Occurrence rates(%)for total sample**	**Unadjusted values**		**Adjusted values**	
		**Continuous age**		**Continuous age**	
		**Odds ratio**	**CI**	**Odds ratio**	**CI**
Difficulty concentrating	45.9	.96+	.95-.97	.97+	.96-.99
Pain	64.1	.97+	.96-.98	.97**	.96-.99
Lack of energy	74.7	.98**	.96-.99	.99	.97-1.01
Cough	37.6	1.00	.98-1.01	1.00	.98-1.02
Feeling nervous	32.4	.98**	.96-.99	.98*	.97-.99
Dry mouth	42.8	1.00	.99-1.01	1.01	.99-1.03
Nausea	31.2	.98**	.97-.99	.98*	.97-.99
Feeling drowsy	58.7	.99	.97-1.00	.99	.98-1.01
Numbness/tingling in hands/feet	31.9	.99	.97-1.00	.99	.98-1.01
Difficulty sleeping	53.3	.96+	.95-.98	.97**	.96-.99
Feeling bloated	24.8	.97+	.95-.98	.97+	.95-.98
Problems with urination	29.5	1.03+	1.02-1.05	1.01	.99-1.03
Vomiting	13.8	.98*	.97-.99	.97*	.95-.99
Shortness of breath	34.6	.99	.98-1.00	.99	.97-1.00
Diarrhea	21.8	1.00	.99-1.02	1.00	.98-1.02
Feeling sad	40.5	.95+	.94-.97	.96+	.94-.97
Sweats	37.4	.98**	.96-.99	.98*	.96-.99
Worrying	43.5	.95+	.94-.96	.95+	.94-.97
Problems with sexual interest	28.3	.97+	.95-.98	.95+	.93-.97
Itching	28.0	.98*	.97-.99	.99	.98-1.01
Lack of appetite	33.9	.99	.97-1.00	.99	.98-1.01
Dizziness	23.8	.99	.98-1.01	.99	.98-1.01
Difficulty swallowing	15.7	1.00	.98-1.02	1.00	.98-1.02
Feeling irritable	40.8	.96+	.95-.97	.96+	.95-.98
Mouth sores	13.7	1.00	.98-1.02	1.01	.99-1.03
Changes in the way food tastes	30.5	1.00	.99-1.02	1.01	.99-1.03
Weight loss	23.1	.99	.98-1.01	1.00	.98-1.02
Hair loss	20.7	.98*	.97-.99	.99	.97-1.01
Constipation	32.2	1.00	.99-1.01	1.00	.98-1.01
Swelling of arms or legs	16.4	1.01	.99-1.03	1.02	1.00-1.04
I don’t look like myself	27.3	.97+	.95-.98	.98*	.96-.99
Changes in skin	28.2	.96+	.94-.97	.98**	.96-.99

Abbreviation: CI, confidence interval.

*p < .05, **p < .01, +p < .001.

As shown in Table 3, after controlling for significant covariates, differences in occurrence rates between the two age groups were found for 15 (46.9%) of the 32 MSAS symptoms. Older patients reported significantly lower occurrence rates for difficulty concentrating, pain, feeling nervous, nausea, feeling drowsy, difficulty sleeping, feeling bloated, vomiting, feeling sad, sweats, worrying, problems with sexual interest, feeling irritable, I don’t look like myself, and changes in skin.

**Table 3:** **Differences in symptom OCCURRENCE rates between younger** (<** 60**, **n** = **263**) **and older** (≥** 60**, **n** = **330**) **patients**

**Symptom**	**Occurrence rates(%)by age group**		**Unadjusted values**		**Adjusted values**	
			**Older age**		**Older age**	
	<**60**	≥**60**	**Odds ratio**	**CI**	**Odds ratio**	**CI**
Difficulty concentrating	57.0	37.0	.44+	.32-.62	.58**	.40-.85
Pain	73.0	57.0	.49+	.35-.69	.51**	.34-.75
Lack of energy	81.7	69.1	.50+	.34-.74	.69	.44-1.08
Cough	40.3	35.5	.81	.58-1.14	.89	.60-1.32
Feeling nervous	39.5	26.7	.56**	.39-.79	.61*	.41-.91
Dry mouth	43.7	42.1	.94	.68-1.30	1.10	.75-1.62
Nausea	38.0	25.8	.57**	.40-.80	.52**	.34-.79
Feeling drowsy	65.0	53.6	.62**	.45-.87	.68*	.47-.99
Numbness/tingling in hands/feet	36.1	28.5	.70*	.50-.99	.76	.51-1.14
Difficulty sleeping	63.9	44.8	.46+	.33-.64	.55**	.38-.80
Feeling bloated	31.9	19.1	.50+	.35-.73	.51**	.33-.78
Problems with urination	21.3	36.1	2.09+	1.44-3.02	1.18	.76-1.83
Vomiting	17.5	10.9	.58*	.36-.92	.41**	.23-.72
Shortness of breath	37.6	32.1	.78	.56-1.10	.73	.48-1.11
Diarrhea	21.7	21.8	1.01	.68-1.49	.86	.54-1.35
Feeling sad	52.1	31.2	.42+	.30-.58	.46+	.31-.67
Sweats	45.2	31.2	.55+	.39-.77	.60**	.41-.88
Worrying	57.8	32.1	.35+	.25-.48	.39+	.27-.56
Problems with sexual interest	35.7	22.4	.52+	.36-.75	.34+	.22-54
Itching	34.2	23.0	.58**	.40-.83	.72	.47-1.08
Lack of appetite	38.0	30.6	.72	.51-1.01	.68	.45-1.02
Dizziness	27.0	21.2	.73	.50-1.06	.71	.46-1.10
Difficulty swallowing	16.3	15.2	.91	.59-1.43	.92	.55-1.56
Feeling irritable	51.7	32.1	.44+	.32-.62	.47+	.32-.69
Mouth sores	14.8	12.7	.84	.52-1.34	.91	.54-1.54
Changes in the way food tastes	31.2	30.0	.95	.67-1.34	1.09	.71-1.68
Weight loss	23.2	23.0	.99	.68-1.46	1.10	.69-1.73
Hair loss	23.6	18.5	.74	.49-1.09	.90	.55-1.49
Constipation	33.8	30.9	.87	.62-1.24	.82	.55-1.22
Swelling of arms or legs	16.3	16.4	1.00	.65-1.55	1.10	.66-1.82
I don’t look like myself	35.4	20.9	.48+	.34-.70	.64*	.42-.98
Changes in skin	38.0	20.3	.42+	.29-.60	.62*	.41-.94

Abbreviation: CI, confidence interval.

*p < .05, **p < .01, +p < .001.

### Differences in symptom severity ratings between older and younger patients

As shown in Table 4, after controlling for significant covariates, differences in severity scores were found for 6 (18.9%) of the 32 MSAS symptoms. Older patients reported significantly lower severity scores for dry mouth, feeling drowsy, feeling sad, sweats, worrying, and I don’t look like myself.

**Table 4:** **Differences in SEVERITY ratings between younger** (<** 60**, **n** = **263**) **and older** (≥** 60**, **n** = **330**) **patients**

**Symptom**	**Sample size**	**Age group**	**Mean score**	**Severity ratings†(%)**				**Unadjusted values**			**Adjusted values**		
								**Older age**			**Older age**		
				**1**	**2**	**3**	**4**	**OR**	**CI**	**p**-**value**	**OR**	**CI**	**p**-**value**
Difficulty concentrating	149	<60	1.67	44.3	45.0	10.1	0.7	.80	.50-1.28	.353	.78	.46-1.32	.354
	115	≥60	1.61	50.4	40.0	7.8	1.7						
Pain	192	<60	2.08	24.5	47.9	22.9	4.7	1.08	.74-1.58	.674	.97	.64-1.49	.898
	181	≥60	2.12	24.3	45.3	24.3	6.1						
Lack of energy	214	<60	2.15	15.4	57.5	23.8	3.3	.91	.64-1.31	.622	.81	.55-1.21	.311
	217	≥60	2.13	21.7	49.8	22.1	6.5						
Cough	102	<60	1.74	46.1	36.3	15.7	2.0	1.04	.63-1.73	.870	.86	.47-1.55	.609
	110	≥60	1.75	44.5	38.2	14.5	2.7						
Feeling nervous	103	<60	1.83	35.9	47.6	13.6	2.9	.81	.47-1.40	.452	.81	.42-1.54	.514
	85	≥60	1.78	40.0	48.2	5.9	5.9						
Dry mouth	110	<60	2.06	28.2	41.8	25.5	4.5	.64	.40-1.03	.068	.54	.32-.91	.020
	131	≥60	1.89	38.2	41.2	14.5	6.1						
Nausea	99	<60	1.99	31.3	42.4	22.2	4.0	.64	.37-1.10	.107	.62	.33-1.14	.123
	81	≥60	1.80	43.2	37.0	16.0	3.7						
Feeling drowsy	167	<60	1.95	24.6	58.1	15.6	1.8	.68	.45-1.03	.068	.61	.38-.96	.033
	169	≥60	1.82	32.5	55.0	10.7	1.8						
Numbness/tingling in hands/feet	94	<60	1.85	36.2	45.7	14.9	3.2	.90	.53-1.55	.710	.79	.42-1.52	.484
	91	≥60	1.85	41.8	38.5	13.2	6.6						
Difficulty sleeping	168	<60	2.23	14.9	54.8	23.2	7.1	.87	.56-1.33	.512	.67	.42-1.09	.108
	141	≥60	2.16	17.0	54.6	24.1	4.3						
Feeling bloated	82	<60	1.91	30.5	51.2	14.6	3.7	.77	.40-1.47	.429	.77	.36-1.66	.510
	55	≥60	1.82	36.4	49.1	10.9	3.6						
Problems with urination	54	<60	1.96	33.3	42.6	18.5	5.6	1.14	.61-2.11	.685	1.10	.56-2.18	.777
	110	≥60	2.01	26.4	52.7	14.5	6.4						
Vomiting	43	<60	1.91	41.9	32.6	18.6	7.0	.90	.38-2.14	.811	.51	.17-1.54	.232
	29	≥60	1.79	37.9	44.8	17.2	0.0						
Shortness of breath	97	<60	1.78	40.2	45.4	10.3	4.1	1.52	.90-2.58	.116	1.27	.71-2.29	.423
	99	≥60	2.00	33.3	42.4	15.2	9.1						
Diarrhea	54	<60	1.93	24.1	61.1	13.0	1.9	.67	.33-1.35	.263	.62	.27-1.42	.258
	63	≥60	1.81	36.5	49.2	11.1	3.2						
Feeling sad	137	<60	1.99	30.7	43.8	21.2	4.4	.43	.26-.72	.001	.43	.24-.76	.003
	93	≥60	1.66	46.2	46.2	3.2	4.3						
Sweats	117	<60	2.14	23.9	44.4	25.6	6.0	.54	.33-.91	.019	.56	.31-.997	.049
	95	≥60	1.86	34.7	46.3	16.8	2.1						
Worrying	152	<60	2.10	20.4	58.6	11.8	9.2	.43	.26-.72	.001	.52	.30-.90	.020
	101	≥60	1.77	38.6	49.5	7.9	4.0						
Problems with sexual interest	92	<60	2.50	22.8	31.5	18.5	27.2	1.43	.82-2.52	.212	1.12	.57-2.19	.745
	69	≥60	2.72	18.8	24.6	21.7	34.8						
Itching	88	<60	1.86	38.6	43.2	11.4	6.8	.84	.46-1.52	.562	.89	.46-1.75	.745
	69	≥60	1.78	42.0	43.5	8.7	5.8						
Lack of appetite	99	<60	2.00	27.3	49.5	19.2	4.0	.96	.57-1.64	.886	.77	.42-1.43	.409
	94	≥60	2.00	27.7	51.1	14.9	6.4						
Dizziness	69	<60	1.54	55.1	37.7	5.8	1.4	.84	.43-1.65	.615	.61	.27-1.39	.241
	64	≥60	1.48	59.4	34.4	4.7	1.6						
Difficulty swallowing	40	<60	2.03	27.5	45.0	25.0	2.5	.53	.23-1.21	.133	.39	.14-1.10	.074
	43	≥60	1.79	37.2	51.2	7.0	4.7						
Feeling irritable	135	<60	1.77	40.7	42.2	16.3	0.7	.82	.50-1.34	.425	.65	.37-1.15	.142
	98	≥60	1.68	41.8	49.0	8.2	1.0						
Mouth sores	37	<60	1.86	43.2	35.1	13.5	8.1	.55	.23-1.34	.188	.52	.19-1.43	.205
	35	≥60	1.60	60.0	22.9	14.3	2.9						
Change in way food tastes	79	<60	2.06	27.8	48.1	13.9	10.1	.81	.46-1.43	.476	1.05	.57-1.93	.883
	91	≥60	1.96	31.9	47.3	14.3	6.6						
Weight loss	60	<60	1.67	48.3	38.3	11.7	1.7	.87	.45-1.69	.674	.60	.28-1.30	.196
	67	≥60	1.63	52.2	35.8	9.0	3.0						
Hair loss	61	<60	2.38	27.9	26.2	26.2	19.7	.97	.50-1.88	.921	.77	.36-1.61	.482
	52	≥60	2.37	32.7	23.1	19.2	25.0						
Constipation	87	<60	2.38	17.2	40.2	29.9	12.6	.67	.39-1.14	.139	.74	.42-1.32	.308
	96	≥60	2.19	24.0	43.8	21.9	10.4						
Swelling of arms or legs	42	<60	2.19	33.3	31.0	19.0	16.7	.92	.43-1.98	.837	.86	.36-2.04	.734
	47	≥60	2.09	25.5	44.7	25.5	4.3						
I don’t look like myself	91	<60	2.14	24.2	47.3	18.7	9.9	.52	.28-.95	.033	.49	.25-.96	.037
	62	≥60	1.82	43.5	32.3	22.6	1.6						
Changes in skin	99	<60	2.07	24.2	53.5	13.1	9.1	.63	.34-1.16	.140	.80	.41-1.56	.510
	61	≥60	1.89	37.7	42.6	13.1	6.6						

Abbreviations: CI, confidence interval; OR, odds ratio.

†Severity ratings = slight (1), moderate (2), severe (3), very severe (4).

### Differences in symptom frequency ratings between older and younger patients

As shown in Table 5, after controlling for significant covariates, differences in frequency scores were found for 4 (12.5%) of the 32 MSAS symptoms. Older patients reported significantly lower frequency scores for feeling nervous, dry mouth, nausea, and feeling bloated.

**Table 5:** **Differences in FREQUENCY ratings between younger** (<** 60**, **n** = **263**) **and older** (≥** 60**, **n** = **330**) **patients**

**Symptom**	**Sample size**	**Age group**	**Mean score**	**Frequency ratings† (%)**				**Unadjusted values**			**Adjusted values**		
								**Older age**			**Older age**		
				**1**	**2**	**3**	**4**	**OR**	**CI**	**p**-**value**	**OR**	**CI**	**p**-**value**
Difficulty concentrating	150	<60	2.19	22.7	44.0	25.3	8.0	.78	.50-1.21	.269	.76	.46-1.25	.277
	122	≥60	2.08	24.6	50.8	16.4	8.2						
Pain	192	<60	2.64	13.0	33.3	30.2	23.4	.96	.67-1.38	.829	.95	.63-1.44	.825
	188	≥60	2.62	14.9	32.4	28.7	23.9						
Lack of energy	215	<60	2.82	5.1	30.2	42.3	22.3	.88	.62-1.23	.446	.77	.53-1.13	.179
	228	≥60	2.74	10.5	32.0	30.3	27.2						
Cough	106	<60	2.12	26.4	41.5	25.5	6.6	.98	.61-1.59	.937	.72	.42-1.25	.246
	117	≥60	2.12	26.5	42.7	23.1	7.7						
Feeling nervous	104	<60	2.13	22.1	48.1	24.0	5.8	.57	.33-.97	.038	.52	.27-.99	.046
	88	≥60	1.92	35.2	45.5	11.4	8.0						
Dry mouth	115	<60	2.66	13.9	26.1	40.0	20.0	.66	.42-1.03	.070	.52	.32-.85	.009
	139	≥60	2.44	20.9	31.7	30.2	17.3						
Nausea	100	<60	2.23	23.0	40.0	28.0	9.0	.55	.32-.94	.028	.54	.29-.99	.047
	85	≥60	1.95	34.1	43.5	15.3	7.1						
Feeling drowsy	171	<60	2.40	11.7	44.4	36.3	7.6	.82	.56-1.22	.338	.65	.42-1.01	.058
	176	≥60	2.34	11.9	52.3	25.6	10.2						
Numbness/tingling in hands/feet	95	<60	2.63	16.8	36.8	12.6	33.7	1.08	.65-1.81	.771	.94	.52-1.72	.846
	94	≥60	2.68	20.2	23.4	24.5	31.9						
Difficulty sleeping	168	<60	2.70	9.5	32.1	37.5	20.8	.88	.59-1.32	.541	.64	.41-1.02	.058
	147	≥60	2.63	9.5	32.7	42.9	15.0						
Feeling bloated	84	<60	2.39	22.6	32.1	28.6	16.7	.54	.29-.98	.042	.49	.24-.99	.046
	63	≥60	2.06	28.6	47.6	12.7	11.1						
Problems with urination	56	<60	2.39	21.4	33.9	28.6	16.1	1.52	.84-2.73	.164	1.29	.68-2.46	.438
	119	≥60	2.61	11.8	33.6	37.0	17.6						
Vomiting	46	<60	1.87	39.1	41.3	13.0	6.5	.85	.38-1.93	.705	.46	.15-1.38	.165
	36	≥60	1.81	44.4	36.1	13.9	5.6						
Shortness of breath	99	<60	2.17	19.2	52.5	20.2	8.1	1.30	.78-2.14	.311	.97	.55-1.70	.916
	106	≥60	2.35	25.5	34.0	20.8	19.8						
Diarrhea	57	<60	2.12	22.8	47.4	24.6	5.3	.56	.29-1.10	.094	.47	.21-1.03	.059
	72	≥60	1.89	29.2	55.6	12.5	2.8						
Feeling sad	137	<60	2.05	28.5	42.3	24.8	4.4	.99	.62-1.59	.978	.79	.46-1.36	.386
	103	≥60	2.08	27.2	47.6	15.5	9.7						
Sweats	119	<60	2.39	16.8	37.0	36.1	10.1	.66	.41-1.07	.093	.64	.37-1.12	.121
	103	≥60	2.21	22.3	44.7	22.3	10.7						
Worrying	152	<60	2.41	9.9	52.0	25.0	13.2	.59	.36-.94	.028	.63	.37-1.08	.094
	106	≥60	2.20	22.6	48.1	16.0	13.2						
Problems with sexual interest	93	<60	2.86	15.1	23.7	21.5	39.8	1.07	.61-1.86	.821	1.04	.53-2.02	.916
	74	≥60	2.89	17.6	17.6	23.0	41.9						
Itching	90	<60	2.38	14.4	46.7	25.6	13.3	.72	.41-1.27	.254	.71	.38-1.33	.289
	76	≥60	2.21	23.7	40.8	26.3	9.2						
Lack of appetite	100	<60	2.44	18.0	39.0	24.0	19.0	1.44	.88-2.38	.149	1.16	.66-2.05	.598
	101	≥60	2.64	18.8	24.8	29.7	26.7						
Dizziness	71	<60	1.82	39.4	40.8	18.3	1.4	.96	.52-1.78	.902	.78	.38-1.61	.502
	70	≥60	1.81	38.6	45.7	11.4	4.3						
Difficulty swallowing	43	<60	2.23	30.2	32.6	20.9	16.3	.87	.41-1.81	.701	.56	.23-1.38	.207
	50	≥60	2.14	32.0	34.0	22.0	12.0						
Feeling irritable	136	<60	1.99	30.1	44.1	22.1	3.7	.78	.48-1.25	.300	.67	.38-1.17	.161
	105	≥60	1.88	33.3	47.6	17.1	1.9						
Mouth sores	39	<60	2.21	38.5	28.2	7.7	25.6	.73	.33-1.63	.447	.75	.29-1.93	.553
	42	≥60	2.02	47.6	23.8	7.1	21.4						
Change in way food tastes	80	<60	2.54	17.5	31.3	31.3	20.0	1.11	.65-1.88	.709	1.16	.65-2.08	.616
	98	≥60	2.60	16.3	33.7	23.5	26.5						
Weight loss	59	<60	2.07	22.0	55.9	15.3	6.8	.76	.40-1.44	.400	.56	.27-1.16	.120
	75	≥60	1.99	34.7	41.3	14.7	9.3						
Hair loss	62	<60	2.85	16.1	22.6	21.0	40.3	1.02	.53-1.95	.953	.89	.43-1.84	.745
	60	≥60	2.85	21.7	15.0	20.0	43.3						
Constipation	89	<60	2.53	14.6	40.4	22.5	22.5	.82	.49-1.38	.455	.87	.49-1.53	.626
	102	≥60	2.42	15.7	45.1	20.6	18.6						
Swelling of arms or legs	43	<60	2.67	23.3	23.3	16.3	37.2	1.04	.51-2.15	.906	.91	.39-2.12	.832
	54	≥60	2.70	24.1	20.4	16.7	38.9						
I don’t look like myself	92	<60	2.67	15.2	29.3	28.3	27.2	.79	.45-1.39	.420	.84	.45-1.57	.587
	69	≥60	2.54	20.3	27.5	30.4	21.7						
Changes in skin	100	<60	2.77	15.0	24.0	30.0	31.0	.69	.39-1.20	.188	.97	.52-1.82	.929
	67	≥60	2.54	23.9	25.4	23.9	26.9						

Abbreviations: CI, confidence interval; OR, odds ratio.

†Frequency ratings = rarely (1), occasionally (2), frequently (3), almost constantly (4).

### Differences in symptom distress ratings between older and younger patients

As shown in Table 6, after controlling for significant covariates, differences in distress scores were found for 14 (43.8%) of the 32 MSAS symptoms. Older patients reported significantly lower distress scores for pain, lack of energy, cough, dry mouth, nausea, feeling drowsy, difficulty sleeping, feeling bloated, feeling sad, sweats, worrying, hair loss, I don’t look like myself, and changes in skin.

**Table 6:** **Differences in DISTRESS ratings between younger** (<** 60**, **n** = **263**) **and older** (≥** 60**, **n** = **330**) **patients**

**Symptom**	**Sample size**	**Age group**	**Mean score**	**Distress ratings†(%)**					**Unadjusted values**			**Adjusted values**		
									**Older age**			**Older age**		
				**0**	**1**	**2**	**3**	**4**	**OR**	**CI**	**p**-**value**	**OR**	**CI**	**p**-**value**
Difficulty concentrating	147	<60	1.52	17.7	38.1	22.4	18.4	3.4	.71	.45-1.10	.124	.82	.50-1.34	.424
	116	≥60	1.33	25.0	37.9	21.6	10.3	5.2						
Pain	192	<60	2.01	6.3	32.3	29.2	19.3	13.0	.63	.43-.90	.012	.65	.43-.98	.041
	183	≥60	1.69	15.8	32.2	26.2	18.0	7.7						
Lack of energy	212	<60	2.04	8.5	26.4	30.2	22.6	12.3	.54	.39-.77	<.001	.57	.39-.83	.004
	220	≥60	1.64	20.5	30.5	22.3	18.6	8.2						
Cough	103	<60	1.39	29.1	28.2	24.3	11.7	6.8	.60	.37-.97	.037	.52	.29-.91	.022
	112	≥60	1.07	42.9	25.0	19.6	7.1	5.4						
Feeling nervous	102	<60	1.70	10.8	38.2	26.5	19.6	4.9	.51	.30-.87	.013	.57	.30-1.06	.075
	85	≥60	1.33	23.5	40.0	22.4	8.2	5.9						
Dry mouth	111	<60	1.45	22.5	37.8	20.7	9.9	9.0	.50	.32-.80	.003	.47	.29-.76	.002
	133	≥60	1.05	42.1	29.3	15.8	7.5	5.3						
Nausea	99	<60	1.84	12.1	32.3	29.3	12.1	14.1	.47	.27-.80	.006	.46	.25-.86	.015
	81	≥60	1.37	33.3	29.6	13.6	13.6	9.9						
Feeling drowsy	165	<60	1.39	21.2	39.4	23.0	12.1	4.2	.53	.36-.79	.002	.52	.34-.80	.003
	167	≥60	1.07	40.1	31.7	13.8	9.6	4.8						
Numbness/tingling in hands/feet	94	<60	1.40	21.3	39.4	20.2	16.0	3.2	.72	.43-1.21	.212	.84	.46-1.56	.586
	91	≥60	1.25	33.0	33.0	16.5	11.0	6.6						
Difficulty sleeping	166	<60	2.01	9.6	29.5	25.9	19.9	15.1	.61	.41-.92	.017	.54	.35-.86	.008
	142	≥60	1.66	19.0	27.5	29.6	16.2	7.7						
Feeling bloated	81	<60	1.85	16.0	24.7	27.2	22.2	9.9	.43	.23-.81	.008	.38	.18-.80	.011
	56	≥60	1.30	19.6	42.9	26.8	8.9	1.8						
Problems with urination	54	<60	1.57	24.1	25.9	25.9	16.7	7.4	1.21	.67-2.19	.530	.91	.47-1.76	.783
	108	≥60	1.70	14.8	32.4	31.5	10.2	11.1						
Vomiting	43	<60	1.63	23.3	32.6	16.3	14.0	14.0	.78	.34-1.79	.553	.58	.21-1.62	.298
	30	≥60	1.43	33.3	20.0	26.7	10.0	10.0						
Shortness of breath	97	<60	1.63	14.4	41.2	21.6	12.4	10.3	.79	.48-1.30	.347	.80	.46-1.39	.427
	100	≥60	1.47	28.0	26.0	24.0	15.0	7.0						
Diarrhea	54	<60	1.59	18.5	38.9	14.8	20.4	7.4	.69	.36-1.34	.274	.61	.28-1.31	.204
	63	≥60	1.32	25.4	33.3	30.2	6.3	4.8						
Feeling sad	137	<60	1.87	8.0	36.5	27.0	17.5	10.9	.47	.29-.77	.003	.49	.28-.85	.011
	94	≥60	1.41	14.9	46.8	24.5	9.6	4.3						
Sweats	117	<60	1.75	17.1	29.1	28.2	12.8	12.8	.49	.30-.80	.005	.56	.32-.98	.040
	95	≥60	1.26	24.2	40.0	24.2	8.4	3.2						
Worrying	151	<60	1.93	7.3	34.4	28.5	17.9	11.9	.45	.28-.72	.001	.57	.34-.96	.033
	101	≥60	1.46	8.9	53.5	23.8	10.9	3.0						
Problems with sexual interest	91	<60	2.08	22.0	19.8	15.4	14.3	28.6	.96	.55-1.66	.875	.89	.46-1.73	.731
	69	≥60	2.03	18.8	20.3	24.6	11.6	24.6						
Itching	88	<60	1.56	15.9	40.9	25.0	8.0	10.2	.82	.46-1.45	.497	1.04	.55-1.95	.908
	69	≥60	1.42	24.6	31.9	27.5	8.7	7.2						
Lack of appetite	99	<60	1.38	27.3	31.3	24.2	10.1	7.1	.85	.51-1.41	.529	.85	.48-1.51	.589
	96	≥60	1.28	32.3	29.2	21.9	11.5	5.2						
Dizziness	69	<60	1.32	23.2	37.7	26.1	10.1	2.9	.84	.45-1.56	.572	.62	.30-1.29	.202
	63	≥60	1.25	23.8	46.0	15.9	9.5	4.8						
Difficulty swallowing	40	<60	1.65	10.0	40.0	30.0	15.0	5.0	.96	.44-2.09	.924	.85	.35-2.06	.724
	43	≥60	1.63	20.9	27.9	23.3	23.3	4.7						
Feeling irritable	135	<60	1.49	21.5	34.8	24.4	11.9	7.4	.79	.49-1.27	.335	.82	.48-1.42	.485
	96	≥60	1.31	18.8	47.9	17.7	14.6	1.0						
Mouth sores	36	<60	1.67	22.2	30.6	19.4	13.9	13.9	.46	.20-1.09	.079	.41	.16-1.03	.058
	35	≥60	1.11	31.4	42.9	11.4	11.4	2.9						
Change in way food tastes	78	<60	1.69	17.9	32.1	25.6	11.5	12.8	.47	.27-.81	.007	.57	.31-1.05	.072
	89	≥60	1.18	32.6	36.0	14.6	14.6	2.2						
Weight loss	61	<60	1.05	45.9	26.2	13.1	6.6	8.2	.67	.35-1.29	.232	.51	.24-1.09	.082
	68	≥60	0.78	54.4	26.5	8.8	7.4	2.9						
Hair loss	61	<60	1.74	19.7	29.5	21.3	16.4	13.1	.50	.26-.97	.041	.48	.23-.99	.048
	55	≥60	1.29	43.6	20.0	9.1	18.2	9.1						
Constipation	87	<60	2.09	13.8	23.0	25.3	16.1	21.8	.68	.41-1.14	.145	.74	.42-1.29	.285
	95	≥60	1.80	23.2	25.3	15.8	20.0	15.8						
Swelling of arms or legs	42	<60	1.64	28.6	23.8	19.0	11.9	16.7	.96	.46-2.01	.913	1.11	.46-2.67	.819
	48	≥60	1.58	27.1	25.0	18.8	20.8	8.3						
I don’t look like myself	91	<60	1.89	11.0	36.3	19.8	18.7	14.3	.32	.17-.58	<.001	.30	.15-.58	<.001
	64	≥60	1.14	34.4	34.4	17.2	10.9	3.1						
Changes in skin	99	<60	1.76	11.1	36.4	29.3	12.1	11.1	.41	.23-.76	.004	.51	.27-.98	.043
	62	≥60	1.26	38.7	24.2	17.7	11.3	8.1						

Abbreviations: CI, confidence interval; OR, odds ratio.

†Distress ratings = not at all (0), a little bit (1), somewhat (2), quite a bit (4), very much (5).

### Differences in MSAS subscale and total scores between younger and older patients

As shown in Table 7, after controlling for significant covariates, older patients reported significantly lower scores on all of the MSAS subscales, as well as on the MSAS total score.

**Table 7:** **Differences in subscale and total scores on the Memorial Symptom Assessment Scale** (**MSAS**) **between younger** (<**60**, **n** = **263**) **and older** (≥** 60**, **n** = **330**) **patients**

**Scale**	**Unadjusted scores**			**Adjusted scores**		
	**Mean(Standard deviation)**			**Mean(Standard error)**		
	**<60 years**	≥ **60 years**	**p**-**value**	**<60 years**	≥ **60 years**	**p**-**value**
Physical subscale	.92 (.63)	.70 (.58)	<.0001	.90 (.04)	.70 (.03)	<.0001
Psychological subscale	1.15 (.84)	.67 (.69)	<.0001	1.10 (.05)	.70 (.04)	<.0001
Global Distress Index	1.17 (.74)	.78 (.70)	<.0001	1.13 (.04)	.81 (.04)	<.0001
Total MSAS score	.84 (.50)	.60 (.46)	<.0001	.81 (.03)	.60 (.03)	<.0001

### Differences in rankings of symptoms with highest occurrence, severity, frequency, and distress ratings between older and younger patients

Table 8 provides a summary of the differences in the rankings of the various symptom dimensions (i.e., occurrence, severity, frequency, distress) for the top 10 symptoms between older and younger patients. For symptom occurrence, the four most prevalent symptoms (i.e., lack of energy, pain, feeling drowsy, difficulty sleeping) were the same for both age groups. Eight of 10 symptoms (80%) were in the top 10 for both age groups. Feeling sad and sweats were unique to younger patients. Problems with urination, cough, and shortness of breath were unique to older patients.

**Table 8:** **Differences between younger** (<** 60**, **n** = **263**) **and older** (≥** 60**, **n** = **330**) **patients in rankings of symptoms with the highest occurrence**, **severity**, **frequency**, **and distress ratings**

		**OCCURRENCE RATINGS**				
Rank		< 60		≥ 60	
	Symptom	% of patients	Symptom	% of patients	
1	Lack of energy	81.7	Lack of energy	69.1	
2	Pain	73.0	Pain	57.0	
3	Feeling drowsy	65.0	Feeling drowsy	53.6	
4	Difficulty sleeping	63.9	Difficulty sleeping	44.8	
5	Worrying	57.8	Dry mouth	42.1	
6	Difficulty concentrating	57.0	Difficulty concentrating	37.0	
7	Feeling sad	52.1	Problems with urination	36.1	
8	Feeling irritable	51.7	Cough	35.5	
9	Sweats	45.2	Shortness of breath	32.1	
10	Dry mouth	43.7	Worrying	32.1	
			Feeling irritable	32.1	
**SEVERITY RATINGS**					
Rank	Symptom	Mean score	Symptom	Mean score	
1	Problems with sexual interest	2.50	Problems with sexual interest	2.72	
2	Hair loss	2.38	Hair loss	2.37	
3	Constipation	2.38	Constipation	2.19	
4	Difficulty sleeping	2.23	Difficulty sleeping	2.16	
5	Swelling of arms or legs	2.19	Lack of energy	2.13	
6	Lack of energy	2.15	Pain	2.12	
7	Sweats	2.14	Swelling of arms or legs	2.09	
8	I don’t look like myself	2.14	Problems with urination	2.01	
9	Worrying	2.10	Shortness of breath	2.00	
10	Pain	2.08	Lack of appetite	2.00	
**FREQUENCY RATINGS**					
Rank	Symptom	Mean score	Symptom	Mean score	
1	Problems with sexual interest	2.86	Problems with sexual interest	2.89	
2	Hair loss	2.85	Hair loss	2.85	
3	Lack of energy	2.82	Lack of energy	2.74	
4	Changes in skin	2.77	Swelling of arms or legs	2.70	
5	Difficulty sleeping	2.70	Numbness/tingling in hands/feet	2.68	
6	Swelling of arms or legs	2.67	Lack of appetite	2.64	
7	I don’t look like myself	2.67	Difficulty sleeping	2.63	
8	Dry mouth	2.66	Pain	2.62	
9	Pain	2.64	Problems with urination	2.61	
10	Numbness/tingling in hands/feet	2.63	Changes in taste	2.60	
**DISTRESS RATINGS**					
Rank	Symptom	Mean score	Symptom	Mean score	
1	Constipation	2.09	Problems with sexual interest	2.03	
2	Problems with sexual interest	2.08	Constipation	1.80	
3	Lack of energy	2.04	Problems with urination	1.70	
4	Pain	2.01	Pain	1.69	
5	Difficulty sleeping	2.01	Difficulty sleeping	1.66	
6	Worrying	1.93	Lack of energy	1.64	
7	I don’t look like myself	1.89	Difficulty swallowing	1.63	
8	Feeling sad	1.87	Swelling of arms or legs	1.58	
9	Feeling bloated	1.85	Shortness of breath	1.47	
10	Nausea	1.84	Worrying	1.46	

For symptom severity, the four most severe symptoms (i.e., problems with sexual interest, hair loss, constipation, difficulty sleeping) were the same for both age groups. Seven of 10 symptoms (70%) were in the top 10 for both age groups. Sweats, I don’t look like myself, and worrying were unique to younger patients. Problems with urination, shortness of breath, and lack of appetite were unique to older patients.

For symptom frequency, the three most frequent symptoms (i.e., problems with sexual interest, hair loss, lack of energy) were the same for both age groups. Seven of 10 symptoms (70%) were in the top 10 for both age groups. Dry mouth, I don’t look like myself, and changes in skin were unique to younger patients. Problems with urination, lack of appetite, and changes in taste were unique to older patients.

For symptom distress, two symptoms (i.e., problems with sexual interest, constipation) were ranked as the first or second most distressing symptom in the two age groups. Six of 10 symptoms (60%) were in the top 10 for both age groups. Nausea, feeling bloated, feeling sad, and I don’t look like myself were unique to younger patients. Problems with urination, shortness of breath, difficulty swallowing, and swelling of arms or legs were unique to older patients.

## Discussion

This study is the first to evaluate for differences in multiple dimensions of the symptom experience (i.e., occurrence, severity, frequency, and distress) between older and younger adults undergoing active treatment for cancer. The majority of the age differences were found in ratings of symptom occurrence with older patients reporting significantly lower occurrence rates for 15 (46.9%) of the MSAS symptoms. While fewer age-related differences were found in ratings of symptom severity, frequency, and distress, for younger and older patients who reported a specific symptom, a similar pattern was found across all three dimensions, namely when differences occurred older patients reported significantly lower ratings.

While this secondary analysis provides interesting and important data on age-related differences in various dimensions of the symptom experience in a very large sample of oncology patients, several limitations need to be acknowledged before our findings are placed within the context of an extremely limited literature. First, detailed information was not available on the exact number or specific types of comorbidities these patients experienced. In addition, specific details on the doses of CTX and RT received by these patients were not collected for these studies. Despite these limitations, findings from this initial analysis can be used to generate testable hypotheses for future research.

For almost 50% of the MSAS symptoms, older patients reported significantly lower occurrence rates. However, eight of the eleven symptoms with the highest occurrence rates were identical in the two age groups (i.e., lack of energy, pain, feeling drowsy, difficulty sleeping, dry mouth, difficulty concentrating, worrying, feeling irritable). Consistent with previous studies of multiple symptoms [15,35-39], these eight symptoms are among the most common symptoms reported by oncology patients regardless of cancer diagnosis, stage of disease, and/or cancer treatment. Since these eight symptom occurrence rates exhibit a similar distribution pattern in both age groups, one plausible hypothesis for the significantly lower occurrence rates for six of these symptoms in older patients (no difference in lack of energy or dry mouth) is that older patients received lower doses of CTX or RT [3,12,14,40]. An equally plausible hypothesis is that older patients received comparable doses of therapy. However, because of age-related changes in a number of biological processes [41] and/or a variety of psychosocial factors [42,43], older patients reported lower symptom occurrence rates. For example, age-related changes may occur in the hypothalamic-adrenal-pituitary axis (HPA) that mediate the occurrence and severity of the most common cancer-related symptoms [44].

Another plausible hypothesis for the lower symptom occurrence rates in older patients is that older persons may experience a “response shift” in their perception of symptoms. A “response shift” is defined as an age-related psychological shift that represents a change in a person’s internal framework for the assessment of experiences [45]. This concept was first used in oncology to describe changes over time in QOL [46]. In the context of older patients’ reports of symptoms, these individuals may have experienced an internal reconceptualization of their symptom experience based on their lifetime experience with symptoms or their experience with symptoms from other chronic medical conditions [45]. In addition, based on studies of barriers to pain assessment and management in older adults [47], older patients tend to under-report pain because: they view it as a normal part of aging; they are concerned that if they report pain it will distract their clinician from treating their cancer; they are fearful about additional diagnostic tests and their associated costs; or they are worried about additional treatments (e.g., opioid analgesics for pain) and associated adverse events [11,48,49]. It is plausible that these same barriers contribute to under-reporting of other symptoms in older adults. Cough and shortness of breath were among the top most frequently occurring symptoms only in the older age group. In contrast, feeling sad and sweats were unique to the younger age group. These differences in patterns of occurrence warrant investigation in future studies.

For older and younger patients who reported a specific symptom, severity ratings for the majority of the symptoms were mild to moderate. For all but six symptoms (i.e., dry mouth, feeling drowsy, feeling sad, sweats, worrying, I don’t look like myself), no age-related differences in severity scores were reported (see Table 3). In addition to the hypotheses stated above, several potential explanations for the lack of differences in symptom severity ratings warrant consideration. If younger patients received higher doses of RT and/or CTX than older patients, one would expect higher symptom severity scores in the younger group. One potential explanation for the equivalent symptom severity scores is that younger patients received more aggressive symptom management than older patients. This hypothesis is supported by studies that found that older patients receive lower doses of opioid analgesics than younger patients with comparable pain severity scores [50,51].

In terms of the occurrence rates for specific symptoms (see Table 8), seven of the ten symptoms with the highest severity ratings were the same in both age groups (i.e., problems with sexual interest, hair loss, constipation, difficulty sleeping, swelling of arms or legs, lack of energy, pain). A similar pattern for the most severe symptoms across age groups suggests that these symptoms are common across cancer treatments. The age differential in terms of actual severity ratings may be related to dose reductions in the elderly or due to other biological or psychological mechanisms described above. Similar hypotheses could be proposed to explain the age-related differences in the symptom frequency ratings.

While a “response shift” in older persons’ perceptions of their symptoms is a plausible explanation for the lower occurrence rates for over 50% of the MSAS symptoms, one might ask why this same hypothesis does not seem to apply for the other dimensions of the symptom experience. One reason that a “response shift” may not be as evident in patients’ ratings of symptom severity, frequency, and distress is that the age group comparisons for these three dimensions were done with only patients who reported the symptom. To verify this hypothesis, differences in symptom severity scores between older and younger patients were re-analyzed with the inclusion of patients who did not report each symptom (i.e., symptom severity scores ranged from 0 to 4 instead of from 1 to 4). In these analyses, compared to younger patients, older patients reported significantly lower severity scores for: difficulty concentrating, pain, lack of energy, feeling nervous, nausea, feeling drowsy, difficulty sleeping, feeling bloated, vomiting, feeling sad, problems with sexual interest, feeling irritable, worrying, I don’t look like myself, and changes in skin. This finding suggests that in older patients who develop a symptom as a result of their cancer or its treatment, they experience it with a similar severity as younger patients. The reasons why some older patients do and do not experience cancer symptoms warrants investigation in future studies.

Besides occurrence, the largest number of age-related differences were found in the symptom distress ratings (i.e., for 43.8% of the symptoms, older patients reported significantly lower ratings). Symptom distress is defined as the degree or amount of physical or mental upset, anguish, or suffering experienced from a specific symptom [26,52]. In one review [39], out of 22 studies that assessed multiple symptoms in cancer patients, only seven (31.8%) assessed distress. Of those seven, three used the MSAS [39]. While no studies were found that evaluated for age differences in MSAS symptom distress ratings, the symptoms with the highest distress ratings in our study are comparable to those found in other studies that used the MSAS [53-55].

As shown in Table 8, six of the ten most distressing symptoms were the same in both age groups (i.e., constipation, problems with sexual interest, lack of energy, pain, difficulty sleeping, worrying). Problems with urination, difficulty swallowing, swelling of the arms or legs, and shortness of breath were among the top most distressing symptoms in the older age group. In contrast, I don’t look like myself, feeling sad, feeling bloated, and nausea were unique to the younger age group. These differences in distress patterns warrant additional research.

Of note, no age-related differences were found in symptom distress ratings for constipation and problems with sexual interest. These two symptoms which occurred in approximately 30% of the patients in both age groups warrant a careful assessment because when they do occur, they are severe, occur frequently, and are distressing not only to older patients, but to younger patients as well.

A surprising finding in this study was that a significantly higher percentage (26%) of older patients reported being fully active compared to younger patients (15.8%). While older individuals in the general population are more likely to report poorer functional status than younger individuals [56], the higher functional status in the older patients in this study may be attributed to lower recruitment rates among the oldest old. An alternative explanation is that younger patients received higher doses of CTX or RT that had a negative impact on their functional status.

Despite the limitations enumerated earlier in the discussion, findings from this study provide valuable information to guide clinical practice and research. As shown in Table 8, across all four symptom dimensions, the most common, severe, frequent, and distressing symptoms are similar for both age groups. What differs is the magnitude estimation for each dimension, with older persons reporting lower rates of occurrence, severity, frequency, and distress for some symptoms. Because the age-related differences in symptom severity, frequency, and distress scores were small, additional research is warranted within each age group to determine the impact of each dimension of a symptom on younger and older patients’ functional status and QOL.

The findings summarized in Table 8 provide new information on symptoms with the highest occurrence, severity, frequency and distress ratings in both age groups. This information can be used by clinicians to guide their multidimensional symptom assessments of older oncology patients. Future research needs to focus on a detailed evaluation of patient (i.e., phenotypic and genotypic characteristics) and clinical characteristics that explain the age differences in the various dimensions of the symptom experience described in this study.

## Conclusions

This study is the first to evaluate for differences in multiple dimensions of symptom experience in older oncology patients. For almost 50% of the MSAS symptoms, older patients reported significantly lower occurrence rates. While fewer age-related differences were found in ratings of symptom severity, frequency, and distress, a similar pattern was found across all three dimensions. Future research needs to focus on a detailed evaluation of patient and clinical characteristics (i.e., type and dose of treatment) that explain the differences in symptom experience identified in this study.

## Abbreviations

CTX: Chemotherapy; ECOG: Eastern Cooperative Oncology Group; FPS: Fatigue, Pain, and Sleep; HPA: Hypothalamic-adrenal-pituitary axis; MSAS: Memorial Symptom Assessment Scale; QOL: Quality of life; RT: Radiation therapy; UCSF: University of California, San Francisco.

## Competing interests

The authors declare that there are no competing interests.

## Authors’ contributions

JC drafted the manuscript, and participated in the interpretation of the data. SP combined the datasets, conducted data analyses, and contributed to the interpretation of the data, and the critical revision of the manuscript. BC conducted analyses and participated in the critical revision of the manuscript. HS contributed to the analysis, the interpretation of the data, and critical revision of the manuscript. KA, BA, VB, JM, and LD contributed to the interpretation of the data, and the critical revision of the manuscript, and CR participated in the critical revision of the manuscript. PY and CM conceived the concept and study design, coordinated the study, and contributed to the interpretation, drafting, and critical revision of the manuscript. All authors read and approved the final manuscript.

## Pre-publication history

The pre-publication history for this paper can be accessed here:

http://www.biomedcentral.com/1471-2407/13/6/prepub

## References

[B1] Jemal A Bray F Center MM Ferlay J Ward E Forman D Global cancer statistics CA Cancer J Clin 2011 61 69 90 10.3322/caac.20107 21296855

[B2] Balducci L Supportive care in elderly cancer patients Curr Opin Oncol 2009 21 310 317 10.1097/CCO.0b013e32832b4f25 19412099

[B3] Townsley C Pond G Peloza B Kok J Naidoo K Dale D Herbert C Holowaty E Straus S Siu L Analysis of treatment practices for elderly cancer patients in Ontario, Canada J Clin Oncol 2005 23 3802 3810 10.1200/JCO.2005.06.742 15923574

[B4] Townsley G Beck S Watkins J “Learning to live with it”: coping with the transition to cancer survivorship in older adults J Aging Stud 2007 21 93 106 10.1016/j.jaging.2006.08.003

[B5] Langer CJ Clinical evidence on the undertreatment of older and poor performance patients who have advanced non-small-cell lung cancer: Is there a role for targeted therapy in these cohorts? Clin Lung Cancer 2011 12 272 279 10.1016/j.cllc.2011.02.001 21704567

[B6] Dale DC Poor prognosis in elderly patients with cancer: the role of bias and undertreatment J Support Oncol 2003 1 suppl 2 11 17 15346995

[B7] Bastiaannet E Portielje JEA van de Velde CJH de Craen AJM van der Velde S Kuppen PJK van der Geest LGM Janssen-Heijnen MLG Dekkers OM Westendorp RGJ Lack of survival gain for elderly women with breast cancer Oncologist 2011 16 415 423 10.1634/theoncologist.2010-0234 21406470 PMC3228128

[B8] Kagan SH Ageism in cancer care Sem Oncol Nurs 2008 24 246 253 10.1016/j.soncn.2008.08.004 19000598

[B9] Kroenke CH Rosner B Chen WY Kawachi I Colditz GA Holmes MD Functional impact of breast cancer by age at diagnosis J Clin Oncol 2004 22 1849 1856 10.1200/JCO.2004.04.173 15143077

[B10] Cheung WY Le LW Gagliese L Zimmermann C Age and gender differences in symptom intensity and symptom clusters among patients with metastatic cancer Support Care Cancer 2011 19 417 423 10.1007/s00520-010-0865-2 20333411

[B11] Carreca I Balducci L Cancer chemotherapy in the older cancer patient Urol Oncol 2009 27 633 642 10.1016/j.urolonc.2009.08.006 19879474

[B12] Brunello A Loaldi E Balducci L Dose adjustment and supportive care before and during treatment Cancer Treat Rev 2009 35 493 498 10.1016/j.ctrv.2009.04.009 19493625

[B13] Kumar A Soares H Balducci L Djulbegovic B Treatment tolerance and efficacy in geriatric oncology: a systematic review of phase III randomized trials conducted by five National Cancer Institute-sponsored cooperative groups J Clin Oncol 2007 25 1272 1276 10.1200/JCO.2006.09.2759 17401017

[B14] Rose J O'Toole E Dawson N Thomas C Connors A Jr Wenger N Phillips R Hamel M Cohen H Lynn J Age differences in care practices and outcomes for hospitalized patients with cancer J Am Geriatr Soc 2000 48 S25 S32 10809453 10.1111/j.1532-5415.2000.tb03137.x

[B15] Mohile SG Heckler C Fan L Mustian K Jean-Pierre P Usuki K Sprod L Janelsins M Purnell J Peppone L Age-related differences in symptoms and their interference with quality of life in 903 cancer patients undergoing radiation therapy J Geriatr Oncol 2011 2 225 232 10.1016/j.jgo.2011.08.002 22888384 PMC3415272

[B16] Degner L Sloan J Symptom distress in newly diagnosed ambulatory cancer patients and as a predictor of survival in lung cancer J Pain Symptom Manage 1995 10 423 431 10.1016/0885-3924(95)00056-5 7561224

[B17] Yan H Sellick K Symptoms, psychological distress, social support, and quality of life of Chinese patients newly diagnosed with gastrointestinal cancer Cancer Nurs 2004 27 389 399 15525867 10.1097/00002820-200409000-00009

[B18] Avis NE Crawford S Manuel J Quality of life among younger women with breast cancer J Clin Oncol 2005 23 3322 3330 10.1200/JCO.2005.05.130 15908646

[B19] Ellis J Lin J Walsh A Lo C Shepherd FA Moore M Li M Gagliese L Zimmermann C Rodin G Predictors of referral for specialized psychosocial oncology care in patients with metastatic cancer: the contributions of age, distress, and marital status J Clin Oncol 2009 27 699 705 10.1200/JCO.2007.15.4864 19114706

[B20] Graves KD Arnold SM Love CL Kirsh KL Moore PG Passik SD Distress screening in a multidisciplinary lung cancer clinic: prevalence and predictors of clinically significant distress Lung Cancer 2007 55 215 224 10.1016/j.lungcan.2006.10.001 17084483 PMC1857305

[B21] Politi MC Enright TM Weihs KL The effects of age and emotional acceptance on distress among breast cancer patients Support Care Cancer 2007 15 73 79 10.1007/s00520-006-0098-6 16816961

[B22] Goldzweig G Hubert A Walach N Brenner B Perry S Andritsch E Baider L Gender and psychological distress among middle-and older-aged colorectal cancer patients and their spouses: an unexpected outcome Crit Rev Oncol Hematol 2009 70 71 82 10.1016/j.critrevonc.2008.07.014 18762432

[B23] Weiss T Weinberger MI Holland J Nelson C Moadel A Falling through the cracks: a review of psychological distress and psychosocial service needs in older Black and Hispanic patients with cancer J Geriatr Oncol 2012 3 2 163 173 10.1016/j.jgo.2011.12.001

[B24] Yabroff KR Lamont EB Mariotto A Warren JL Topor M Meekins A Brown ML Cost of care for elderly cancer patients in the United States J Nat Cancer Inst 2008 100 630 641 10.1093/jnci/djn103 18445825

[B25] Smith BD Smith GL Hurria A Hortobagyi GN Buchholz TA Future of cancer incidence in the United States: burdens upon an aging, changing nation J Clin Oncol 2009 27 2758 2765 10.1200/JCO.2008.20.8983 19403886

[B26] Portenoy RK Thaler HT Kornblith AB McCarthy Lepore J Friedlander-Klar H Kiyasu E Sobel K Coyle N Kemeny N Norton L The memorial symptom assessment scale: an instrument for the evaluation of symptom prevalence, characteristics and distress Eur J Cancer 1994 30 1326 1336 10.1016/0959-8049(94)90182-1 7999421

[B27] Kim H Barsevick A Tulman L Predictors of the intensity of symptoms in a cluster in patients with breast cancer J Nurs Scholar 2009 41 158 165 10.1111/j.1547-5069.2009.01267.x PMC2884278 19538700

[B28] Brant J Beck S Dudley W Cobb P Pepper G Miaskowski C Symptom trajectories in posttreatment cancer survivors Cancer Nurs 2010 34 67 77 20885306 10.1097/NCC.0b013e3181f04ae9

[B29] Hopwood P Haviland J Mills J Sumo G The impact of age and clinical factors on quality of life in early breast cancer: an analysis of 2208 women recruited to the UK START Trial (Standardisation of Breast Radiotherapy Trial) Breast 2007 16 241 251 10.1016/j.breast.2006.11.003 17236771

[B30] Gagliese L Jovellanos M Zimmermann C Shobbrook C Warr D Rodin G Age-related patterns in adaptation to cancer pain: a mixed method study Pain Med 2009 10 1050 1061 10.1111/j.1526-4637.2009.00649.x 19594849

[B31] Gagliese L Melzack R Age-related differences in the qualities but not the intensity of chronic pain Pain 2003 104 597 608 10.1016/S0304-3959(03)00117-9 12927632

[B32] Zubrod CG Schneiderman M Frei E Brindley C Gold GL Shnider B Appraisal of methods for the study of chemotherapy of cancer in man: comparative therapeutic trial of nitrogen mustard and triethylene thiophosphoramide J Chron Dis 1960 11 7 33 10.1016/0021-9681(60)90137-5

[B33] Verger E Salamero M Conill C Can Karnofsky performance status be transformed to the Eastern Cooperative Oncology Group scoring scale and vice versa? Eur J Cancer 1992 28 1328 1330 10.1016/0959-8049(92)90510-9 1515244

[B34] Hamilton W Lancashire R Sharp D Peters TJ Cheng K Marshall T The risk of colorectal cancer with symptoms at different ages and between the sexes: a case–control study BMC Med 2009 7 1 17 10.1186/1741-7015-7-17 19374736 PMC2675533

[B35] Stark LL Tofthagen C Visovsky C McMillan SC The symptom experience of patients with cancer J Hosp Palliat Nurs 2012 14 61 70 10.1097/NJH.0b013e318236de5c 22639548 PMC3358129

[B36] Dodd MJ Miaskowski C Paul SM Symptom clusters and their effect on the functional status of patients with cancer Oncol Nurs Forum 2001 28 465 470 11338755

[B37] Dodd MJ Miaskowski C Lee KA Occurrence of symptom clusters JNCI Monographs 2004 32 76 78 10.1093/jncimonographs/lgh008 15263044

[B38] Kim EJ Dodd M Aouizerat B Jahan T Miaskowski C A review of the prevalence and impact of multiple symptoms in oncology patients J Pain Symptom Manage 2009 37 715 736 10.1016/j.jpainsymman.2008.04.018 19019626 PMC2688644

[B39] Gilbertson-White S Aouizerat BE Jahan T Miaskowski C A review of the literature on multiple symptoms, their predictors, and associated outcomes in patients with advanced cancer Palliat Support Care 2011 9 81 102 10.1017/S147895151000057X 21352621

[B40] Hamaker M Schreurs W van Slooten H Uppelschoten J Smorenburg C Trends in breast cancer treatment in the elderly at a breast cancer outpatient clinic: guidelines followed better Ned Tijdsch Geneeskd 2009 153 A562 19930741

[B41] McLachlan AJ Pont LG Drug metabolism in older people—a key consideration in achieving optimal outcomes with medicines J Gerontol A Biol Sci Med Sci 2012 67 175 180 21835808 10.1093/gerona/glr118

[B42] Kahana E Kahana B Kelley-Moore J Adams SA Hammel R Kulle D Brown JA King C Toward advocacy in cancer care for older adults: survivors have cautious personal actions but bold advice for others J Am Geriatr Soc 2009 57 S269 S271 20122027 10.1111/j.1532-5415.2009.02509.x

[B43] Lenze EJ Wetherell JL A lifespan view of anxiety disorders Dialogues Clin Neurosci 2011 13 381 399 22275845 10.31887/DCNS.2011.13.4/elenze PMC3263387

[B44] Bower JE Low CA Moskowitz JT Sepah S Epel E Benefit finding and physical health: positive psychological changes and enhanced allostasis Soc Personal Psychol Compass 2008 2 223 244 10.1111/j.1751-9004.2007.00038.x

[B45] Costanzo ES Ryff CD Singer BH Psychosocial adjustment among cancer survivors: findings from a national survey of health and well-being Health Psycho 2009 28 147 156 10.1037/a0013221 PMC2668871 19290706

[B46] Schwartz CE Sprangers MAG Methodological approaches for assessing response shift in longitudinal health-related quality-of-life research Soc Sci Med 1999 48 1531 1548 10.1016/S0277-9536(99)00047-7 10400255

[B47] Herr K Titler MG Schilling ML Marsh JL Xie X Ardery G Clarke WR Everett LQ Evidence-based assessment of acute pain in older adults: current nursing practices and perceived barriers Clin J Pain 2004 20 331 340 10.1097/00002508-200409000-00008 15322440

[B48] Pain(PDQ) http://www.cancer.gov/cancertopics/pdq/supportivecare/pain/HealthProfessional/page1

[B49] Ferrell BA Pain management in elderly people J Am Geriatr Soc 1991 39 64 73 1670940 10.1111/j.1532-5415.1991.tb05908.x

[B50] Urban D Cherny N Catane R The management of cancer pain in the elderly Crit Rev Oncol Hematol 2010 73 176 183 10.1016/j.critrevonc.2009.03.008 19372049

[B51] Delgado-Guay MO Bruera E Management of pain in the older person with cancer. Part 1 Oncology 2008 22 148 152 18409661

[B52] Rhodes VA Watson PM Symptom distress–the concept: past and present Sem Oncol Nurs 1987 3 242 247 10.1016/S0749-2081(87)80014-1 3321273

[B53] McMillan SC Small BJ Symptom distress and quality of life in patients with cancer newly admitted to hospice home care Oncol Nurs Forum 2002 29 1421 1428 10.1188/02.ONF.1421-1428 12432413

[B54] Peters L Sellick K Quality of life of cancer patients receiving inpatient and home-based palliative care J Adv Nurs 2006 53 524 533 10.1111/j.1365-2648.2006.03754.x 16499673

[B55] Rodin G Zimmermann C Rydall A Jones J Shepherd FA Moore M Fruh M Donner A Gagliese L The desire for hastened death in patients with metastatic cancer J Pain Symptom Manage 2007 33 661 675 10.1016/j.jpainsymman.2006.09.034 17531909

[B56] Sergi G Sarti S Mosele M Ruggiero E Imoscopi A Miotto F Bolzetta F Inelmen EM Manzato E Coin A Changes in healthy elderly women's physical performance: a 3-year follow-up Exp Gerontol 2011 46 929 933 10.1016/j.exger.2011.08.008 21884781

